# COVID-19 pandemic impact on neurologic emergencies: a single-center retrospective cohort study

**DOI:** 10.11604/pamj.2022.41.255.33897

**Published:** 2022-03-29

**Authors:** Maria Gavriilaki, Eleni Karlafti, Maria Moschou, Konstantinos Notas, Marianthi Arnaoutoglou, Georgia Kaiafa, Christos Savopoulos, Vasilios Kimiskidis

**Affiliations:** 1 First Department of Neurology, American Hellenic Educational Progressive Association (AHEPA), University Hospital, School of Medicine, Aristotle University of Thessaloniki, Thessaloniki, Greece,; 2 First Department of Propaedeutic and Internal Medicine, American Hellenic Educational Progressive Association (AHEPA), University Hospital, Aristotle University of Thessaloniki, Thessaloniki, Greece,; 3 Laboratory of Clinical Neurophysiology, American Hellenic Educational Progressive Association (AHEPA) University Hospital, Aristotle University of Thessaloniki, Thessaloniki, Greece

**Keywords:** Emergency service, hospital, nervous system diseases, COVID-19, SARS-CoV-2

## Abstract

**Introduction:**

COVID-19 pandemic caused a major disruption to healthcare system. A year after COVID-19 outbreak, the question remains to what extent the lockdowns changed the volume of non-infected patients who were admitted to the Neurologic Department (ND). To determine the impact of the pandemic´s first year on a tertiary ND.

**Methods:**

non-infected patients admitted to ND between March 2020 and February 2021 were examined. A control group was generated for the same time interval starting from March 2019. Primary outcomes were the number of patients presenting with neurologic complaint who were admitted to the hospital and the diagnosis type. Secondary outcomes were hospitalization length and patients´ outcome.

**Results:**

overall, 816 patients (49.4% females) were admitted during the predetermined periods. Median age was 55 years. Median length of hospitalization was six days. We observed a 47.2% reduction in our department´s admissions during pandemic (n=282). None of the examined variables (type of neurologic diagnosis, age, gender, hospitalization length and outcome) changed significantly during pandemic. However, the number of patients admitted during the pandemic with a diagnosis categorized as “other” was statistically significant lower compared to the year before COVID-19 (p=0.007). Hospitalization length was associated only with patients´ age.

**Conclusion:**

our study examined for the first-time the consequences of the first year of COVID-19 pandemic on ND admissions. COVID-19 outbreak resulted in decreased admissions. Delays in seeking medical consultation for urgent or undiagnosed neurologic conditions require rigorous long-term monitoring to fully understand the impact of COVID-19 pandemic on patients with neurologic diseases.

## Introduction

The first confirmed case of severe acute respiratory syndrome coronavirus 2 (SARS-CoV-2)-induced disease (coronavirus disease-2019, COVID-19) was announced in Greece on 26^th^ February 2020. SARS-CoV-2 was first detected in Wuhan, China in December 2019 and was rapidly spread altering everyday life worldwide [1]. Since the COVID-19 pandemic reached Greece, government ordered twice a series of restrictive measures including lockdowns in mid-March and in early November 2020 causing a major disruption to healthcare system. Outpatient clinics and scheduled procedures were suspended, and hospitals were flooded by COVID-19 patients.

Concurrently, physicians and researchers struggled to decipher the pathophysiology and neurologic sequelae of acute COVID-19 disease [2]. Early reports showed that SARS-CoV-2 infection´s first presentation could be a cerebrovascular event or a variety of central and peripheral nervous system (CNS and PNS) manifestations [3,4]. However, the enormous scientific and healthcare interest in COVID-19 along with the increasing public uncertainty and fear surrounding the nonessential visits to the hospitals edged out non-COVID-19 services. Given the extended duration of these restrictive measures there are emerging concerns regarding the long-lasting impact of treatment postponements and delays in seeking medical consultation on non-COVID-19 patients affected by neurologic disorders.

**Aim:** a year after COVID-19 outbreak, the question remains to what extent the lockdowns changed the volume of non-infected patients that admitted to the neurologic department. Therefore, the objective of this single-center retrospective study was to analyze the changes in diagnoses, hospitalization rates, and Emergency Department (ED) admissions at the First Department of Neurology of AHEPA University Hospital of Thessaloniki, the first hospital in the Northern Greece assigned as “COVID-19 hospital”.

## Methods

**Study design:** a single-center retrospective cohort study was conducted in the AHEPA University Hospital of Thessaloniki, a tertiary university hospital and referral center in the Northern Greece.

**Setting, participants and study size:** to examine the impact of pandemic on non-COVID-19 related neurologic disorders and ED admissions, we included all consecutive patients with neurologic complaints, that presented to our ED and admitted at the First Department of Neurology between 1^st^ March 2020 until 28^th^ February 2021. This period is referred to as the during pandemic´. We also examined a control group which was resulted from the same time interval starting from 1^st^ March 2019 until 29^th^ February 2020 (before pandemic´). We excluded all patients with a positive COVID-19 diagnostic test at the time of ED presentation and patients younger than 16-years.

**Variables and data sources:** the patient database was retrieved from ED registrations. We collected data from medical records on demographics, presenting symptom, length of hospitalization, COVID-19 diagnostic test, discharge diagnosis and outcome. We classified neurologic disorders into the following types: i) cerebrovascular diseases (ischemic stroke, intracranial hemorrhage, arterial dissection, subarachnoid hemorrhage, aneurysm, unknown cause); ii) epilepsy; iii) multiple sclerosis and CNS inflammatory disease; iv) movement disorders; v) neuromuscular and PNS disorders (including focal and multifocal neuropathies and cranial nerve palsies); vi) headache disorders; vii) infectious diseases; viii) disorders of consciousness (including transient loss of consciousness and delirium); (ix) CNS tumors; (x) other (dementia and cognitive impairment, spinal cord disorders, sensory loss, dizziness, narcolepsy, toxicities, transient global amnesia, trauma, undetermined).

**Statistical methods and quantitative variables:** data were analyzed using IBM SPSS Statistics (IBM SPSS Statistics for Windows, Version 26.0. Armonk, NY: IBM Corp). Variables examined in this analysis were age, gender, period (“before” or “during pandemic”), diagnosis, length of hospitalization, and outcome. Descriptive analysis was performed using frequencies and percentages for categorical variables, and median/interquartile range (IQR) or mean/standard deviation (SD) whenever appropriate for continuous variables. Kolmogorov-Smirnov test of normality was applied. We performed a univariable analysis using Fisher's exact and the chi-squared test for categorical variables. For continuous variables, the non-parametric Mann-Whitney U-test or Kruskal-Wallis tests were used along with a Bonferroni correction for multiple comparisons. Moreover, multivariable linear and logistic regression analyses were performed for the risk factor assessments of hospitalization length and outcome. The final multivariable models were built using variables with p≤0.2 in the univariate analysis. Significance level was defined at 0.05 and two-tailed. CIs and p values were obtained based on a 5% significance level and all tests were two-sided.

**Bias:** the present single-center retrospective cohort study is reported in accordance with the STROBE (Strengthening the Reporting of Observational studies in Epidemiology) statement [5]. The study was approved by the hospital´s scientific committee on 2^nd^ June 2021, protocol number 298/02.06.21. All patients who admitted at our department signed an informed consent form for personal data processing.

**Ethical considerations:** the study was approved by the hospital´s scientific committee on 2^nd^ June 2021, protocol number 298/02.06.21. All patients admitted at our department signed an informed consent form for personal data processing.

## Results

**Participants:** during March 2019-February 2020 (before pandemic) and March 2020-February 2021 (during pandemic), 534 (65.4%) and 282 (34.6%) patients, respectively, were admitted to the first department of neurology through the ED for the management of one of the aforementioned neurologic diseases.

**Figure 1:**
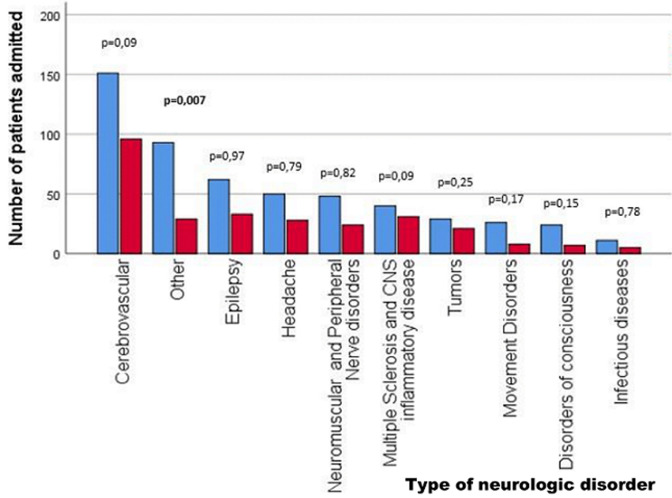
diagnoses of patients admitted to neurologic department before and during COVID-19 pandemic

**Descriptive data:** overall, 816 patients (49.4% females) were admitted during the predetermined periods. Median age was 55 years (range 16-92). Median length of hospitalization was six days (range 1-83). We observed a 47.2% reduction in our department´s admissions during pandemic. Descriptive characteristics of patients admitted during each period are displayed in Table 1. Interestingly, none of the examined variables changed significantly during pandemic (Table 1). However, the number of patients admitted with a minor presenting symptom such as dizziness or sensory loss (categorized as “other” diagnosis) during the pandemic was statistically significant lower compared to the year before the pandemic (p=0.007). Differences among frequencies of neurologic diagnoses leading to patient admission during and before the pandemic are shown in Figure 1.

**Table 1:** characteristics of patients admitted to the neurologic department through the emergency department before and during COVID-19 pandemic

Variables		Before pandemic (N=534)	During pandemic (N=282)	Total (N=816)	p-value^a^
Gender	Male	260	153	413 (50.6%)	0.1
	Female	274	129	403 (49.4%)	
Age (years)		55 (23)	55 (25)	55 (16-92)	0.4
Length of hospitalization (days)		6 (7)	6 (7)	6 (1-83)	0.3
Diagnosis	Cerebrovascular	151 (28.3%)	96 (34%)	247 (30.3%)	0.065
	Epilepsy	62 (11.6%)	33 (11.7%)	95 (11.6%)	0.065
	Multiple sclerosis and CNS inflammatory disease	40 (7.5%)	31 (11%)	71 (8.7%)	0.065
	Movement disorders	26 (4.9%)	8 (2.8%)	34 (4.2%)	0.065
	Neuromuscular and peripheral nerve disorders	48 (9%)	24 (8.5%)	72 (8.8%)	0.065
	Headache	50 (9.4%)	28 (9.9%)	78 (9.6%)	0.065
	Infectious diseases	11 (2.1%)	5 (1.8%)	16 (2%)	0.065
	Disorders of consciousness	24 (4.5%)	7 (2.5%)	31 (3.8%)	0.065
	Tumors	29 (5.4%)	21 (7.5%)	50 (6.1%)	0.065
	Other	93 (17.4%)	29 (10.3%)	122 (15%)	0.065
Outcome	Stable	239	91	330 (40.4%)	0.001
	Amelioration	278	187	465 (57%)	0.001
	In-hospital transfer	1	0	1 (0.1%)	0.001
	Deterioration	9	1	10 (1.2%)	0.001
	Death	7	3	10 (1.2%)	0.001

Continuous variables are presented by median and interquartile range (IQR); ^a^P-values obtained by Mann-Whitney U test for two independent samples for continuous variables and chi-square test or Fisher’s exact test for categoricals

**Outcome data:** the most common diagnosis was cerebrovascular disease presenting in 30.3% of the admitted patients, followed by other diagnoses (15%) and epilepsy (11.6%). As expected, the most observed type of cardiovascular event was ischemic stroke (60.3%). Transient ischemic attack (TIA) occurred in 20.7%, intracranial haemorrhage in 6.5%, aneurysm or arterial dissection or arteriovenous malformation in 2%, cerebral venous thrombosis in 1.2% of patients with a cerebrovascular event. In 9.3% of patients with cerebrovascular events the aetiology was not determined.

**Main results and other analyses:** among various diagnoses age and length of hospitalization differed significantly. Patients presenting with a cerebrovascular event were statistically significant older (p<0.001) than patients with epilepsy, multiple sclerosis and CNS inflammatory disease, headache, and disorders of consciousness. Furthermore, length of hospitalization was statistically significant longer (p<0.001) for patients with cerebrovascular disease than for patients with epilepsy, multiple sclerosis and CNS inflammatory disease, neuromuscular and peripheral nerve disorders, headache, disorders of consciousness and other diagnosis.

Overall, 2.6% of the patients had an unfavourable outcome. Of note, two patients were tested positive for COVID-19 during hospitalization and were transferred at department of infectious diseases. The first one was a 63-year-old female presenting with acute onset focal neurological symptoms mimicking a cerebrovascular event. The second patient was a 21-year-old female who presented with severe headache. Multivariate analyses were performed to examine potential risk factors of hospitalization length and outcome (multivariable linear and logistic regression models, respectively). We controlled all independent variables univariately as potential risk factors. We found that length of hospitalization was associated significantly only with patient´s age (β-coefficient=0.083, 95% CI 0.053, 0.113) meaning that for each year added in patient's age the length of hospitalization is getting longer by almost one day. None of the examined variables significantly affected patient outcome according to our logistic regression analysis.

## Discussion

Emergency services and inpatient care have undergone massive alterations during COVID-19 outbreak. Neurologists adapted to change and reorganized their clinics to provide the highest quality of care without exposing chronic patients to any risk of infection. This retrospective cohort study compares the first year of the COVID-19 pandemic with the same time interval preceding pandemic in 2019 and early 2020. We found a 47.2% reduction in the number of non-infected patients admitted to the First Department of Neurology of AHEPA Hospital. This observation is in line with other cohorts worldwide published during the first months of the pandemic [6,7]. Nevertheless, these early reports present notable limitations considering the limited period studied at the onset of COVID-19 pandemic. We examined the effects of two consecutive lockdowns imposed over one year on the admissions of a neurologic department located in a tertiary COVID-19 referral center. Of note, the only difference observed in the etiology of the admissions was the reduction of patients admitted for other diagnoses depicting public resistance to visit the hospital for minor symptoms. While emergencies requiring hospitalization were reduced, demographic characteristics, length of hospitalization and patient´s outcome remained stable during pandemic. Length of hospitalization was associated only with patients´ age.

Interestingly, only two patients out of 282 admissions had a positive test during hospitalization at out department. This could be attributed to the thorough screening for COVID-19 infection before the admission along with the effective use of personal protective equipment by healthcare staff. Patients who were found positive for COVID-19 in the ED were admitted to specialized isolated departments as hospital protocol suggested from the beginning of this pandemic to mitigate exposure of non-infected patients and healthcare service providers. Moreover, since January 2021 a robust COVID-19 vaccination program has launched in Greece. Healthcare service providers and immunocompromised or patients with chronic neurologic diseases received priority for COVID-19 vaccines. The rapid development of the vaccines and the slowly growing herd immunity surely affected the admissions at our department and will change the course of COVID-19 pandemic.

Our study confirmed that COVID-19 altered everyday function of a neurologic department by prioritizing in-hospital services exclusively for life-threatening neurologic diseases as well as by introducing virtual clinics for the management of non-life-threatening. Several neurologic organizations published guidelines for the management of every type of neurologic disease during the pandemic [8-11]. Treatment and electrodiagnostic studies protocols changed according to the expected risk of severe COVID-19 disease for the patients and the safety of frontline healthcare workers. Some reports weighing benefits and risks of in hospital transmission suggested that intravenous therapies which do not increase the risk of COVID-19 should be rather administered at home than at infusion centers [12]. Nevertheless, other treatments such as spinal muscular atrophy´s intrathecal drug delivery should not be delayed despite the increased risk of COVID-19 exposure at the hospital [13,14]. One example of the COVID-19 impact on management of neurologic diseases are the recommendations published for the use of multiple sclerosis disease-modifying therapies (DMTs) [15]. According to this report, physicians should not initiate treatment with a DMT associated with a high risk of systemic immunosuppression (cladribine, alemtuzumab, autologous hematopoietic stem cell transplantation) or consider an alternative DMT during the COVID-19 pandemic. Furthermore, specific guidance to stroke centers was published for treating stroke patients by administering endovascular treatments [16]. Tenecteplase over alteplase thrombolysis was also considered to be a reasonable alternative to reduce risk of the COVID-19 virus transmission in the ED [17]. Finally, the American Academy of Neuromuscular and Electrodiagnostic Medicine encouraged physicians to carefully prioritize, delay or cancel non-urgent electrodiagnostic studies [18].

Except for the physician´s guidance to avoid any non-life-threatening face-to-face care visit an overall hospital avoidance by public was observed. We found a statistically significant reduction in admissions for other neurologic conditions than the nine main diagnosis types. A possible explanation is likely the message to “stay at home” and the adoption of telemedicine into everyday patient's life to protect people suffering from chronic neurologic disorders. We could only speculate that the incidence of neurologic emergencies did not drop during COVID-19 and the differences derived from the aforementioned alterations in public health policy and public reluctance to visit hospitals. Undoubtedly, the COVID-19 pandemic improved the way we deliver care as it paved the way for the introduction of telemedicine. Telemedicine offered numerous opportunities during the COVID-19 crisis by decreasing non-essential face-to-face visits which could be adopted into routine healthcare when the pandemic is over. For some neurologic conditions, for example epilepsy, telemedicine can be an excellent tool in assessing seizure control, managing treatment's side effects, or making medication adjustments in chronic patients [19]. However, considerable limitations concerning the neurologic physical examination and the management of newly diagnosed patients through telehealth exist. Given the overall benefits of hospital care for some neurologic conditions and the importance of hospital diagnosis for new onset neurologic symptoms for which medical consultation was not sought, the pandemic's collateral impact on non-COVID-19 conditions could not be measured yet. Only future studies with longer follow-up could assess the long-lasting consequences of this pandemic on the prevalence of neurologic diseases and on the management of patients with chronic neurologic disorders.

**Limitations:** the strengths of the present study are the large number of patients included and the long-time intervals assessed. This is the first study comparing the effects of the COVID-19 pandemic on patients presenting to a neurological emergency room and admitted to a neurologic department during the first 12-months. The main limitation is based on the study design. The retrospective single-center cohort setting underlies the possibility of a misclassification bias along with possible unweighted confounding factors. However, even though our department is a large referral tertiary center the generalizability of our findings for different populations and settings should not be assumed. We examined only patient records from ED visits. Thus, we could not assess the number of patients with neurologic diseases who avoided or postponed emergency hospital care or sought care outside the hospital´s ED including private practice, outpatient centers or telemedicine consultations during the first year of COVID-19 pandemic.

## Conclusion

Our retrospective study examined for the first-time the consequences of the first year of COVID-19 pandemic on neurologic admissions. Our data confirm previous observations based on shorter time periods. Further well-designed prospective studies should examine the prevalence of neurologic diseases and the possibly altered structure of neurologic departments during post lockdown period. In hospital care avoidance and delays in seeking medical consultation for emergency or undiagnosed neurologic conditions require rigorous long-term monitoring to fully understand the enormous impact of COVID-19 pandemic on public health.

### What is known about this topic

- COVID-19 pandemic caused a major disruption to healthcare system;
- The enormous scientific and healthcare interest in COVID-19 along with the increasing public uncertainty and fear surrounding the nonessential visits to the hospitals edged out non-COVID-19 services;
- The question remains to what extent the lockdowns changed the volume of non-infected patients who were admitted to the neurologic department.

### What this study adds

- This single-center retrospective cohort study demonstrates that during the first year of COVID-19 outbreak admissions to the neurologic department were decreased by 47.2% and the severity of presenting complaints changed compared to the year before;
- Type of neurologic diagnosis, age, gender, hospitalization length and outcome did not change significantly during COVID-19 outbreak;
- The number of patients admitted during the pandemic with a diagnosis related with minor presenting complaints was statistically significant lower compared to the year before COVID-19.
